# Local interaction in retinal ganglion cell mosaics can generate a consistent spatial periodicity in cortical functional maps

**DOI:** 10.1186/1471-2202-16-S1-P192

**Published:** 2015-12-18

**Authors:** Jaeson Jang, Se-Bum Paik

**Affiliations:** 1 Department of Bio and Brain Engineering, KAIST, Daejeon, 305-701, Republic of Korea

## 

Orientation map is one of the most studied functional maps in visual cortex, but the developmental mechanism of its consistent spatial periodicity is still elusive. Recently, a theoretical model suggested that a moiré interference pattern between ON and OFF retinal ganglion cell (RGC) mosaics can develop a quasi-periodic orientation map, but it is remained unclear how this can explain the constant periodicity of the maps [1]. Here we suggest a developmental model that a simple local interaction in RGCs can generate a consistent spatial periodicity of orientation preference, by inducing (i) a hexagonal lattice structure in ON/OFF RGC mosaics and (ii) a constant alignment angle between them.

First, we introduced a developmental model of a monotypic RGC mosaic to show that a local repulsive interaction can generate a hexagonal structure (Figure 1A). Previously, in the model study of the pairwise interaction point process, it was suggested that a local interaction alone cannot develop a long-range order in the mosaic structure [2]. We assumed a different type of local repulsive interaction that the cell positions can be gradually shifted by a repulsion from the neighbor cells and confirmed that this model can develop a long-range ordered structure that is well fitted to a hexagonal lattice.

**Figure 1:**
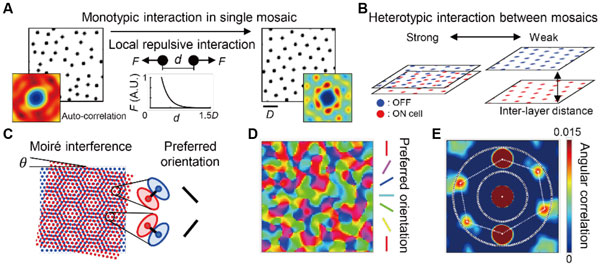
**Simulation of RGC mosaics and orientation map**. **A**. Development of monotypic mosaic and auto-correlation. **B**. Heterotypic interaction depends on inter-layer distance. **C**. Moiré interference pattern of ON/OFF RGC mosaics. **D**. Simulated orentation map. **E**. Hexagonal pattern in auto-correlation of orientation map.

Next, we assumed that there also exists a heterotypic repulsive interaction between ON and OFF RGC mosaics and examined how this can affect the alignment between the two mosaics (Figure 1B). When the inter-layer distance between ON/OFF mosaics was varied within a proper interval, the hexagonal structure was preserved in each mosaic, but the alignment angle (*θ*) between the two mosaics was restricted within a certain range of angles, and this induced a constant spatial periodicity in the ON/OFF interference pattern (Figure 1C). As observed in the moiré interference, we confirmed a consistent hexagonal periodicity in the cortical orientation map that are simulated by statistical wiring model from the developed RGC mosaics (Figure 1D,E) [3].

## Conclusions

Our result suggests that a local repulsive interaction in RGC mosaics can generate a hexagonal structure in ON/OFF RGC mosaics and a restricted alignment between them. The interference between mosaics induces a consistent spatial periodicity in cortical orientation map as predicted by the moiré interference pattern.
